# Comparative genomics of two super-shedder isolates of *Escherichia coli* O157:H7

**DOI:** 10.1371/journal.pone.0182940

**Published:** 2017-08-10

**Authors:** Robab Katani, Rebecca Cote, Indira T. Kudva, Chitrita DebRoy, Terrance M. Arthur, Vivek Kapur

**Affiliations:** 1 Department of Animal Science, The Pennsylvania State University, University Park, Pennsylvania, United States of America; 2 The Huck Institutes of the Life Sciences, The Pennsylvania State University, University Park, Pennsylvania, United States of America; 3 Department of Veterinary and Biomedical Sciences, The Pennsylvania State University, University Park, Pennsylvania, United States of America; 4 Food Safety and Enteric Pathogens Research Unit, National Animal Disease Center, Agricultural Research Service, U.S. Department of Agriculture, Ames, Iowa, United States of America; 5 *E*. *coli* Reference Center, The Pennsylvania State University, University Park, Pennsylvania, United States of America; 6 Roman L. Hruska U.S. Meat Animal Research Center, Agricultural Research Service, U.S. Department of Agriculture, Clay Center, Nebraska, United States of America; Tianjin University, CHINA

## Abstract

Shiga toxin-producing *Escherichia coli* O157:H7 (O157) are zoonotic foodborne pathogens and of major public health concern that cause considerable intestinal and extra-intestinal illnesses in humans. O157 colonize the recto-anal junction (RAJ) of asymptomatic cattle who shed the bacterium into the environment through fecal matter. A small subset of cattle, termed super-shedders (SS), excrete O157 at a rate (≥ 10^4^ CFU/g of feces) that is several orders of magnitude greater than other colonized cattle and play a major role in the prevalence and transmission of O157. To better understand microbial factors contributing to super-shedding we have recently sequenced two SS isolates, SS17 (GenBank accession no. CP008805) and SS52 (GenBank accession no. CP010304) and shown that SS isolates display a distinctive strongly adherent phenotype on bovine rectal squamous epithelial cells. Here we present a detailed comparative genomics analysis of SS17 and SS52 with other previously characterized O157 strains (EC4115, EDL933, Sakai, TW14359). The results highlight specific polymorphisms and genomic features shared amongst SS strains, and reveal several SNPs that are shared amongst SS isolates, including in genes involved in motility, adherence, and metabolism. Finally, our analyses reveal distinctive patterns of distribution of phage-associated genes amongst the two SS and other isolates. Together, the results of our comparative genomics studies suggest that while SS17 and SS52 share genomic features with other lineage I/II isolates, they likely have distinct recent evolutionary histories. Future comparative and functional genomic studies are needed to decipher the precise molecular basis for super shedding in O157.

## Introduction

Shiga toxin-producing *Escherichia coli* O157:H7 (O157) is a major foodborne pathogen that causes severe illnesses in humans worldwide. Symptoms vary from bloody diarrhea with abdominal discomfort to hemorrhagic colitis, and may also include severe life-threatening complications such as hemolytic uremic syndrome (HUS) [1]. Identified as a human pathogen in 1982, the Centers for Disease Control and Prevention (CDC) has estimated that O157 infections cause 73,000 illnesses, 2,200 hospitalizations and 60 deaths annually in the United States, as well as several outbreaks and sporadic infections globally [2].

Asymptomatic cattle are the principal animal reservoir and carry the bacteria predominantly in the terminal recto-anal junction (RAJ) of the gastrointestinal tract [3, 4]. O157 adherence and colonization of the RAJ results in cattle shedding the pathogen in their feces, leading to environmental contamination and transmission of the organism, and ultimately contamination of the food supply and outbreaks of disease in humans. Typically, cattle range from being non-shedders of O157 to shedding 100 CFU/g of feces; however, subgroups of cattle, termed as “super-shedders” (SS) have been found to excrete O157 at levels of ≥10^4^ CFU/g of feces [3, 4]. Several epidemiological modeling studies have suggested that although the number of SS animals on farm is often less than 10%, these animals are responsible for up to 99% of the bacteria shed into the environment [5]. The shedding of this pathogen at several magnitudes higher than normal cattle leads to greater potential of contamination of the surrounding environment and food supply [5]. Epidemiological studies of farms suggest that greater than 96% of O157 isolates originate from the 9% of animals that are super-shedders [6]. Each of the three principal components of the epidemiologic triad—the pathogen, the host, and the environment are likely to contribute to the SS phenotype, and several studies have investigated host and environmental factors associated with super-shedding [3, 6–9]. However, very little is known about the microbial factors that may contribute to super-shedding including the presence of virulence and adherence genes, the ability to form biofilm on biotic and abiotic surfaces, growth and survival rates, nutrient utilization, and other factors affecting overall fitness [4, 8, 9].

We have recently reported the sequence of the complete genomes of two SS strains and shown that SS strains display a distinctive adherence phenotype on rectal squamous epithelial cells recovered from the bovine RAJ [10, 11]. Our current comparative genomics investigations seek to define genomic features that may distinguish SS isolates from others in order to better understand the evolutionary history of SS stains and provide a framework for identifying molecular correlates associated with the SS phenotype in strains of O157.

## Materials and methods

### Bacterial strains

**Fig 1:**
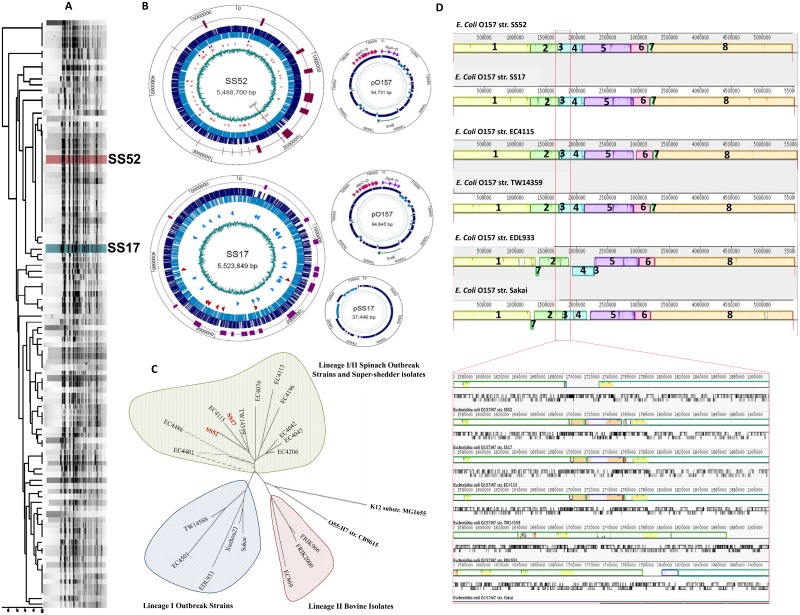
Comparative analysis of SS strains and reference O157 genomes. (A) Dendrogram representing cluster analysis of SS isolates based on PFGE patterns as previously described [12] highlighting the two representative isolates, SS17 and SS52, that have been completely sequenced; (B) Circular genome representations of SS17 and SS52. Larger circles depict chromosomal DNA with smaller circles representing plasmids. The blue circles represent the ORFs and the outer circle (purple) represents the phages in each genome; (C) Cladogram based on whole genome alignment reveals that SS17 and SS52 clusters closely with lineage I/II “spinach” outbreak isolates (EC4115 and TW14359) as compared with lineage I outbreak isolates (Sakai and EDL933) or the bovine lineage II isolates; (D) Whole genome alignments of SS strains with reference O157 strains using progressiveMauve depicting 8 homology blocks of similarity and patterns of divergence among the strains.

SS strains of O157 were isolated and characterized from approximately 3,500 cattle sampled during the summer months over a two-year period in U.S. Midwestern States, as described [12]. The isolates were characterized by phage typing and *Xba*I-based pulsed-field gel electrophoresis (PFGE) (Fig 1A, S1A Fig), and PCR was performed to confirm the presence of genes for O157 antigen, H7 flagella, γ-intimin, and at least one of the Shiga toxin genes as described [13]. Two representatives SS isolates, strains SS17 and SS52 were selected for the study [12]. Both SS17 and SS52 carried phage type 4 and were isolated from supershedding cattle at concentrations of 3.1x10^5^ CFU/rectal swab and 6.8 x 10^5^ CFU/rectal swab, respectively. The PFGE analysis of the SS52 matched the most common PFGE pattern of O157 isolates from human foodborne outbreaks [14]. *E*. *coli* O157 strains Sakai [NC_002695], EDL933 [NC_002655], TW14359 [NC_013008], and EC4115 [NC_011353] were used as reference genomes for comparative analyses [15–18] (Table 1). All bacterial culturing were performed in a BSL-2 facility (IBC # 46638).

**Table 1:** Characteristics of *E*. *coli* O157:H7 isolates.

Isolate	Accession No.	Isolation Source	Outbreak Source	Reference
Sakai	NC_002695	Human	Radish Sprouts	Hayashi, *et al*. 2001
EDL933	NC_002655	Ground Beef	Hamburger	Perna, *et al*. 2001
TW14359	NC_013008	Human	Spinach	Kulasekara, *et al*. 2009
EC4115	NC_011353	Human	Spinach	Eppinger, *et al*. 2011
SS17	CP008805	Bovine Feces	SS Strain	Cote, *et al*. 2015
SS52	CP010304	Bovine Feces	SS Strain	This Study

### Genome sequencing and assembly

Genomic DNA was isolated using QIAGEN DNeasy Blood and Tissue Kit (cat. no. 69504) as previously described [10]. Genome sequencing and assembly procedures have previously been described for SS17 with minor modifications [11] [10]. Genomic DNA from SS52 was submitted to the Genomics Core Facility at The Pennsylvania State University for whole genome shotgun sequencing using the Ion Torrent PGM sequencer (Life Technologies, Grand Island, NY) [19], using a 318 sequencing chip and mate-pair sequencing. A total of 5.4M reads with an average length of 169 bases were obtained with >168-fold coverage [11]. Whole genome restriction optical map was generated using *BamH*I digestion by OpGen, Inc. (Gaithersburg, MD) [20]. Both *de novo* and reference-guided assemblies were performed using DNASTAR SeqMan NGen 3.1 and Lasergene Suite V. 11.1 (Madison, WI) to obtain large contigs, and the genomes closed with a primer walking approach [11]. Circular representations of the genome were generated with GenVision 10 (SS17) and 11(SS52) (DNASTAR, Madison, WI) (Fig 1B).

### Comparative genomics

Complete genomes of *E*. *coli* O157 strains Sakai [NC_002695], EDL933 [NC_002655], TW14359 [NC_013008], EC4115 [NC_011353], and the two SS strains, SS17 [AC_CP008805] and SS52 [AC-CP010304], were evaluated by comparative analyses [10, 11, 15–18]. Whole genome alignments were performed using Mauve and the progressiveMauve algorithm to identify single nucleotide polymorphisms (SNPs), genomic islands, and relative relationships between SS strains and reference strains [21, 22]. A lineage-specific polymorphism assay (LSPA) was performed using primers, described in Yang, *et*. *al* [23], and Sanger sequencing to confirm insertions and deletions. Circular representations of the genome were generated with GenVision 11 for SS52 (DNASTAR, Madison, WI). The whole genome phylogenetic tree was constructed using RealPhy [24] by inputting the Genbank accession IDs for the O157:H7 sequences. The tree builder used was PhyML and was run with 1016 bootstrap iterations (-b 1016), and using the output tree as the Maximum Likelihood tree. The tree was drawn with FigTree V1.4 (Institute of Evolutionary Biology, University of Edinburgh, http://tree.bio.ed.ac.uk/). The phage analysis was performed using PHAST software [25]. BLAST (http://blast.ncbi.nlm.nih.gov/Blast.cgi) was performed for phage alignment including on a pairwise basis.

## Results

### Comparative genomic analysis of SS and reference isolates of O157

We have previously described the complete genome sequence of the prototypical SS strain, SS17 (accession no. CP008805 [10]), and recently completed the sequencing of a second O157 SS strain, SS52 (accession no. CP010304 [11], Fig 1B, S1B Fig). The availability of these high quality full genome sequences provided an opportunity to apply comparative genomic approaches to identify genetic features that may contribute to the SS phenotype.

The analyses showed that the genomes of SS17 and SS52 are similar to reference O157 strains [15–18] (Table 2). The whole genome phylogenetic and LSPA analyses reveal that both SS strains belong to O157 lineage I/II and cluster with isolates of spinach-associated outbreaks, including strains TW14359 and EC4115, and can be differentiated from the prototype lineage I O157 complete genomes of strains Sakai and EDL933, and bovine isolates lineage II (Fig 1C, S1C Fig) [12].

**Table 2:** Genome statistics of SS strains and reference O157 strains.

	SS17	SS52	EC4115	TW14359	Sakai	EDL933
Length of Sequence (Mb)	5.52	5.5	5.57	5.52	5.49	5.53
G+C ratio (%)	50.5	50.5	50.5	50.5	50.5	50.4
Coding DNA Sequences	5442	5655	5608	5555	5504	5587
Protein Coding Region (%)	88.5	88.8	86.8	86.9	86.9	86.5
Average ORF length (bp)	898	862	863	864	868	856
No. rRNA	22	22	22	22	22	22
No. tRNA	107	106	109	108	103	100
No. Plasmids	2	1	2	1	2	1

Alignments of the full genomes of SS17, SS52, and reference O157 strains using the progressiveMAUVE algorithm [21, 22] identified eight distinct blocks of homology, distinct prophage profiles, and two blocks with an inversion and dislocation (Fig 1D, S1D Fig).

Block 1 (Fig 1D, S1D Fig, highlighted in yellow) is a ~1.2Mb section present in all aligned genomes, except for the O157 strain EDL933, where it is 1.3Mb. The increase in the size is due to two insertions in EDL933 including a prophage that is inserted between the ATP dependent protease subunit gene, *clpA* and transposase genes and tRNA-SerW, and insertion of prophage CP-933M between *yccK*, a sulfur transfer protein and tRNA-SerT. The remaining block is well conserved, and begins with *thrA* gene, which is involved in the tryptophan biosynthetic process, and ends with *apg* gene, which plays a role in the late stage of autophagosome formation.

Block 2 (Fig 1D, S1D Fig, highlighted in light green) is a ~500kb region in strains SS17, SS52, EC4115, and TW14359. The area is about 20kb larger in EDL933, due to the insertion of mobile elements, and is rich in several prophages including CP-933N, CP-933O, and CP-933X. Also conserved in this area are genes including *cah*, a calcium-binding and heat-extractable autotransporter protein; *flgA* that is involved in the flagellar biosynthesis; and *fhuE*, an outer membrane receptor for ferric iron uptake. The block starts with *ycdG* gene (uracil permease) and ends with phage related proteins.

Block 3 (Fig 1D, S1D Fig, highlighted in dark blue) is a 2kb region in SS52, ~ 40kb in SS17, EC4115, and TW14359; and ~18 kb in both EDL933 and Sakai strains, and translocated and inverted EDL933 in comparison with the SS strains (SS17 and SS52) and the spinach outbreak strains (TW14359 and EC4115) strains. The primary difference in size results from a ~ 37.5kb insertion in SS17 that includes several transposases and integrases with sequence identity to phage CP-933O. This fragment in SS52 encodes for the putative Rem protein, putative cytoplasmic protein, and *rusA* gene, a Holliday Junction resolvase. The rest of the block is interrupted by phage and related proteins. The block in EC4115, and TW14359 contains genes including exonuclease VIII. In Sakai and EDL933, the area carries phage proteins, an integrase, *lomP*, an outer membrane protein gene and few other hypothetical proteins.

Block 4 (Fig 1D, S1D Fig, highlighted in light blue) is a ~350kb block in all genomes. Both blocks 3 and 4 have been interrupted by the insertion of phages CP-933O, and CP-933P, and the entire section consists of phage related proteins, and phage associated DNA methyl transferase. This block in SS52 contains several phage related proteins, intestinal colonization factors, phage regulators, phage antiterminator Q, non-LEE-encoded type III effector, and several transposases. This block is translocated and inverted in EDL933 genome. In Sakai, the translocation of areas 3 and 4, has led to a smaller section, which mostly contains phage related hypothetical proteins with the section including all phage- related proteins inserted in between both sections. EDL933 carries a similar section as Sakai with similar gene contents; however, area number 3 has translocated to block six.

Block 5 (Fig 1D, S1D Fig, highlighted in purple) is ~ 790kb in SS17, SS52, EC4115, and TW14359 strains, and 720kb in EDL933 and Sakai. This region is mostly conserved among SS17, SS52, EC4115, and TW14359. However, there are two (~5kb and ~2kb) insertions representing remnants of phages in both EDL933 and Sakai as compared to the SS isolates and other strains from lineage 1/II. The phage area contains phage CP-933R, other phages, phage—related proteins, and some transposase, which extend into the next homology block. The entire section is inverted in EDL933 as compared to the rest of the O157 genomes along with the translocation of the area three in other genomes.

Block 6 (Fig 1D, S1D Fig, highlighted in red) is ~ 272kb in SS17, SS52, EC4115, and TW14359 genomes, however, the area is smaller in EDL933 and Sakai, due to phage indels. Genome of EDL933 encodes for a ~ 25 kb CP-933V region that starts from this block with the *intV*, a integrase for prophage CP-933V after *yehV* (transcriptional regulator), and extended into block 5 ending with the gene *yehU* (component sensor protein). Shiga toxin gene stx1A is encoded within this region. This region encodes for many genes that are conserved among all O157 genomes. A few examples of genes that code for metabolism- related proteins in this region are *yoaE* (Putative transport), *edd* (Phosphogluconate dehydratase), *yeaG* (Protein kinase), or *topB* (Topoisomerase III). Gene *yeeJ*, encoding a hypothetical adhesin and an intimin/invasin homolog, is conserved among all genomes except for Sakai. This block also contains a gene encoding an ~4kb bacteriophage lambda -related protein and transposase in the genome of EC4115, not seen in the genomes of the other strains.

Block 7 (Fig 1D, S1D Fig, highlighted in dark green) is a 62kb section conserved among SS17, SS52, EC4115, and TW14359. The region is translocated to block 2 and inverted in both EDL933 and Sakai genomes. The region contains genes that encode for phages, Q933 antiterminator, and portal proteins of BP-933W. The region also contains genes encoding *stx2A2* and *stx2B* subunits in all genomes, and contains tRNA-Arg and prophage BP-933W-related proteins. In all genomes the block is flanked on one end with phage BP-933W integrase, encoding for the Shiga toxin II subunits A and B followed by protein Q protein of the bacteriophage BP-933W (*Q933*). Further analysis reveals that in both SS genomes, EC4115, and TW14359 genomes, the *stx2B* and *stx2A* subunits are followed by three tRNAs, tRNA-Arg, tRNA-Arg, and tRNA-Met, however, in Sakai and EDL933 genomes the three tRNAs are tRNA-Arg, tRNA-Arg, and tRNA-Ile.

Block 8 (Fig 1D, S1D Fig, highlighted in orange) is a 2.2Mb section that is well-conserved in SS17, SS52, EC4115, TW14359, EDL933 and Sakai, and due to the presence of phage and transposases, the block is ~ 35kb larger in Sakai.

Finally, the genomes of SS17, EC4115, and TW14359 encode for a small extra block not observed in SS52, EDL933, or Sakai (data not shown). The block is 821bp in SS17 located between block 5 and 6, and 1771bp located between blocks 3 and 4 in the EC4115 and TW14359 genomes, and encodes for hypothetical proteins.

### Phages are the primary drivers of the recent evolutionary history of SS and other reference O157 isolates

**Fig 2:**
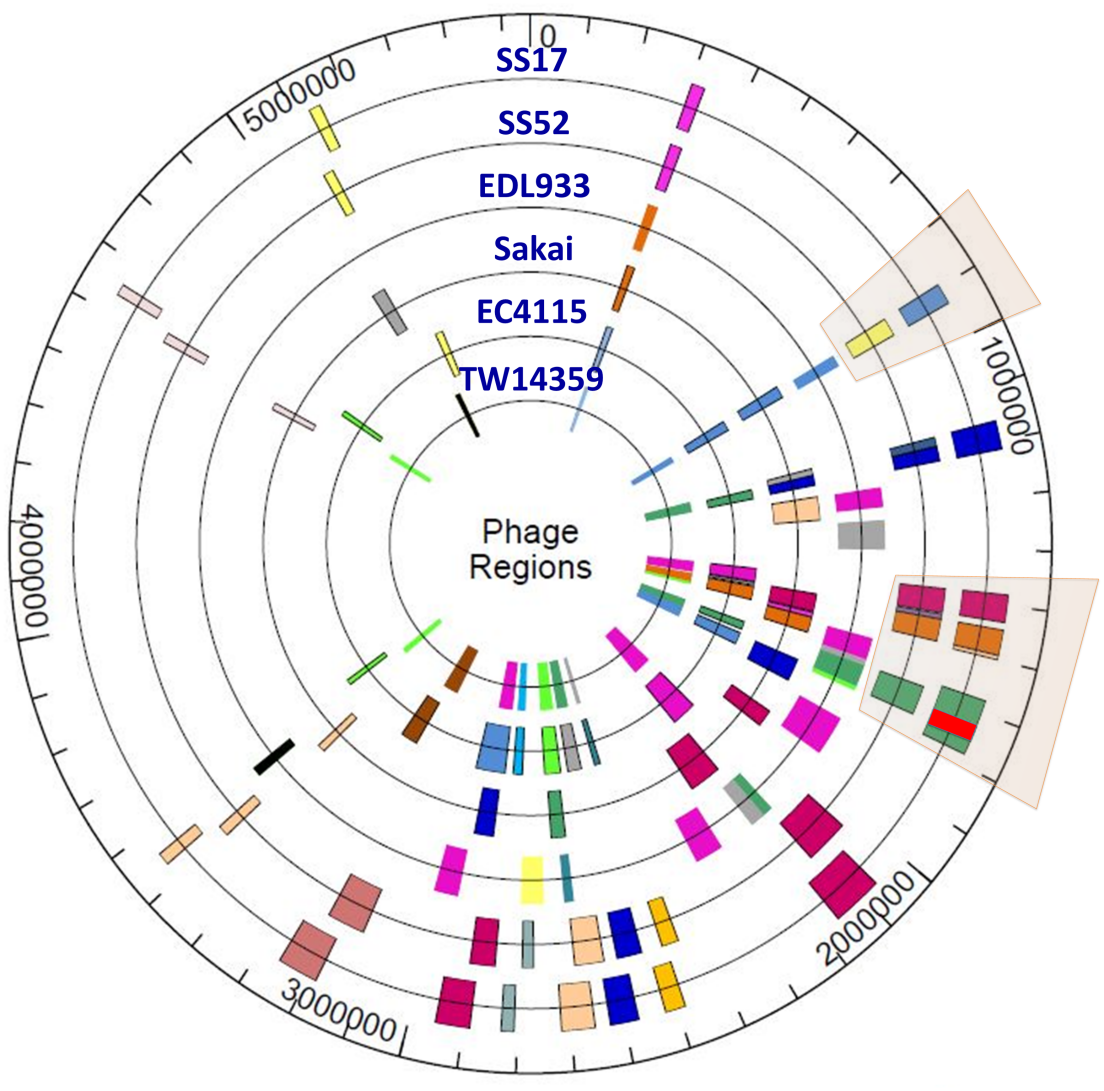
Phages in SS17, SS52, and reference O157 strains. The location and identities of phages were determined in each strain using the Phast server [25]. The color-coded blocks represent phage identity with similar colors showing the same phage on the circular genome. The highlighted area around 1.7MB depicts the insertion of a phage within another phage in SS17 but not SS52. From inner ring: TW14359, EC4115, Sakai, EDL933, SS52, and SS17.

**Fig 3:**
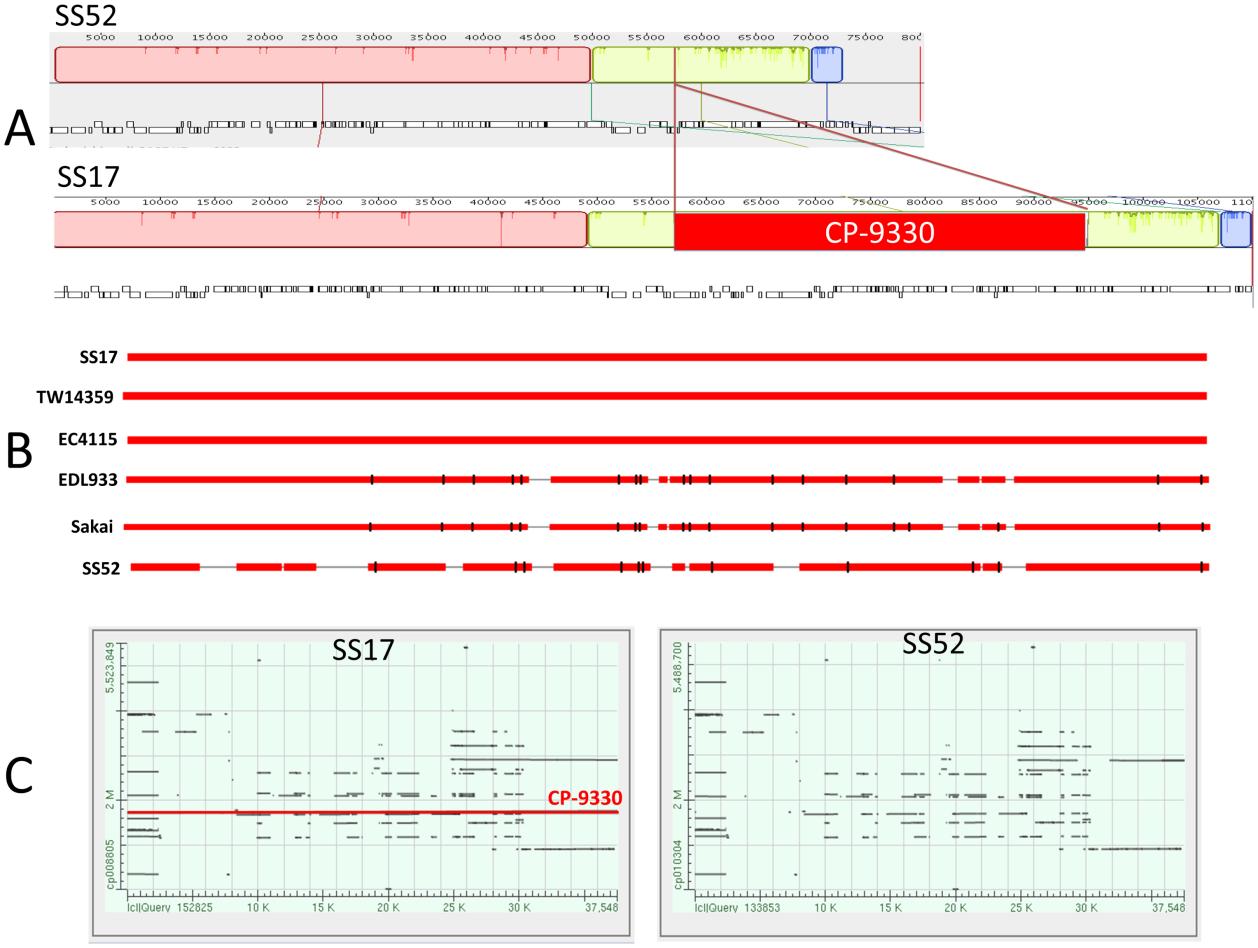
Phage diversity in SS and reference O157 strains. (A) MAUVE alignment of SS17 and SS52 showing the ~37 Kb insertion of CP-933O phage in the SS17 genome; (B) BLAST analysis of the region reveals that SS17, TW14359, and EC4115 all contain the same phage, whereas EDL933, Sakai, and SS52 do not; (C) Dot matrix representation of the results of megaBLAST analysis of the 37.5kb CP-933O region reveals its presence in SS17 (left panel) but not SS52 (right panel).

The genomes of O157 (as well as, other *E*. *coli* strains) are naturally evolving by acquiring new genes, indels, rearrangements, point mutations, and phage-mediated lateral gene transfer. These acquisitions are recognized to be a major driver of the acquisition of new virulence or other traits that contribute to the fitness and adaptation of the pathogen in a particular environment [26–28]. Whole genome comparative analyses of phages in the SS and other reference O157 strains reveal between 17–19 phage regions in each of the genomes and provide strong evidence that phages are the primary drivers of genome scale variation in both SS and other O157 strains (Fig 2; S1 and S2 Tables). SS17 and SS52 phage BLAST analysis indicates that SS17 has an insertion of ~ 37 Kb in the phage region starting from SS17-1718, position 1,726,958 to SS17-1774, position 1,765,158 (Fig 3). SS17, TW14359, and EC4115 share the same uninterrupted phage region, whereas SS52, EDL933, and Sakai have a different pattern. EDL933 and Sakai have a very similar pattern of phage insertion. The region starts with a transposase, and includes genes encoding for prophage CP-933O protein, integrase, and CP-933O encoded exodeoxyribonuclease VIII. It also encodes for antitermination Q and phage portal protein, and finally a terminase. In this region SS17 encodes for 140 CDS and SS52 70 CDS (S3–S5 Tables).

There are some similarities among the SS in this region as well. For instance, SS17, CD # 32 to 69 and SS52, CD # 14 to 51 carry the same pattern of phages, specifically in phage entero HK630_(NC-019723) (Fig 3; S5 Table).

### Analysis of SNPs in the genomes of SS and reference O157 strains

The analyses of SS52 revealed 801 SNPs in SS52 as compared with SS17, 384 SNPs as compared with TW14359, 604 as compared with EC4115, 2136 as compared with Sakai, and 3106 as compared with EDL933, not including ambiguous bases (Table 3). The analyses further identified a total of 167 non-synonymous SNPs (nsSNPs) between the core genomes of SS17 and SS52. Of interest are genes encoding for membrane proteins, such as *yjgN and yebS*; genes involved in the virulence and adhesion such as *eivA*, encoding a type III secretion protein, and a FimD-like (PapC) fimbriae anchoring protein, and *tccP*, the tir-cytoskeleton coupling protein; and interestingly genes involved in the iron acquisition and heme cycle, such as *chuA*, the outer membrane hemoglobin receptor, *chuS*, a heme transport protein, and *afuB*, a ferric iron ABC transporter (S6 Table).

**Table 3:** Polymorphism analysis of SS52 compared with reference O157 genomes.

SNP category	SS17	EC4115	TW14359	Sakai	EDL933
nsSNP	167	152	114	679	866
sSNP	262	295	189	1009	1363
Nonsense	7	5	4	15	13
Homopolymer	139	26	7	57	113
Frameshift	129	18	9	60	143
Frameshift due to homopolymer	56	7	2	26	46
Total SNPs	801	604	384	2136	3106

Although phylogenetic analysis placed SS52 in the same lineage as EC4115 and TW14359, SS52 harbors several strain specific nsSNPs in genes as compared to TW14359 and EC4115 that are involved in the adherence, motility, and cell metabolism. For instance, when compared with TW14359, SS52 harbors nsSNPs in the genes encoding *fimB*, the type 1 fimbriae regulatory protein; *flgA*, encoding flagellar biosynthesis/ motility, and *fhuC*, a ferric hydroxamate transporter, involved in the transport of Fe3+ into O157 cells. As compared to EC4115, SS52 harbors nsSNPs in the genes encoding *flgA* (similar to TW14359), and *tar*, a chemotaxis protein involved in the flagellar regulon. Curiously, compared to EDL933 and SS17, SS52 has a nsSNP in the *tcc*P gene at two different sites. TccP is a tir-cytoskeleton coupling effector protein that plays a role in the production of the “attaching and effacing” (A/E) lesions in the host intestinal mucosa, the hallmark of the O157 pathogenesis and adherence in humans.

## Discussion

Microbial factors leading to super-shedding in O157 are not fully understood. Our previous genomic and phenotypic comparisons of one SS isolate (SS17) showed that SS strains, including SS52, exhibit strong aggregative adherence patterns that are distinctive from the adherence pattern observed in reference O157 strain EDL933 [10]. The adherence characteristics of eight other isolates of SS, including SS52 also revealed the same strong aggregative adherence pattern on the RSE cells as well (S2 Fig) [10]. In order to identify whether there are shared genetic features that contribute to these observed distinctive adherence phenotype in SS strains, we have recently reported the sequence of a second SS isolate, SS52 [11], and have here performed a detailed comparative genomic analyses of SS17 and SS52 strains with other already characterized O157 genomes.

The results of our whole genome comparisons show that SS strains belong to lineage I/II and are clustered with O157 isolates associated with the “spinach” outbreaks of diseases first described in 2009 [17]. Further, our studies reveal that SS17 and SS52 are more closely related to EC4115 and TW14359 strains as compared with EDL933 and Sakai. Based on these analyses, it is tempting to speculate whether other lineage I/II isolates, including EC4115 and TW14359, may also have the potential to represent super-shedder isolates. However, given that the shedding status of the animal from which these two reference strains may have originated is unknown [15, 17], it will be interesting to test whether these two strains share the same adherence phenotype on bovine RAJ stratified squamous epithelial (RSE) that is characteristic of other SS strains [10].

The comparative genomic studies -show that even while the two supershedder isolates of SS17 and SS52 belong to the same genotypic class and display the same adherence phenotype, they are distinct at the genome level, with much of the genomic variation driven by phages and extrachromosomal elements. For instance, SS52 is missing the plasmid pSS17 that harbors a type IV secretion system and several hypothetical genes. This plasmid was likely either recently gained by SS17 or, conversely, lost by SS52 and thus may not have a direct role to play in maintenance of the SS phenotype. It will, however, be interesting in future studies to determine the presence of pSS17 amongst other SS isolates or cure SS17 of pSS17 to formally test its role in the hyper-adherent phenotype seen amongst SS strains.

The overall genomic comparison analyses of both SS genomes with reference genomes indicate that the length of sequences, G+C ratio and number of tRNAs in SS strains are, in general, similar to the reference strains. It is noteworthy that the average ORF length (at 898bp) is longer in the previously described SS17 genome than in reference strains [10]. In contrast, the average ORF length of SS52 (862bp) is consistent with that of reference strains (average 863; range 856–868; Table 2), suggesting that the longer ORF length is particular to SS17 but perhaps not in other SS strains. Interestingly however, at ~ 88.7%, both SS17 and SS52 have a somewhat greater fraction of the genome that is protein coding as compared with the reference strains (average 86.7%; Table 2). While the biological significance of this apparently higher protein-coding region is unknown, we cannot rule out the possibility that this result is merely an artifact of the annotation process that may be resolved by updating the annotation of the reference genomes.

One of the more striking observations resulting from our investigation was the role that phages have played in diversifying the closely related SS isolates. Our studies provide strong evidence that the acquisition and loss of mobile genetic elements are likely the primary drivers of genomic change in SS (and other) O157 strains, and are responsible for the complex patterns of genome scale evolution in the recent evolutionary histories of these isolates. For instance, while SS17 and SS52 are clearly very closely related and share many genomic and phenotypic features including most of the phages, the presence of CP-9330 in SS17, TW14359 and EC4115 clearly differentiates this cluster from SS52 which lacks CP-9330 and is instead similar at this locus to the genomes of EDL933 and Sakai that are Lineage I strains and otherwise genetically and phenotypically distinct. While the role of CP-9330 in enhancing fitness or contributing to virulence of O157 is unknown, these observations suggest that it may not contribute to the SS phenotype, though this hypothesis needs to be formally tested. Together, the results suggest that the patterns of phage evolution drive much of the genome scale variation observed in SS isolates, and this is also evidenced by the distinctive PFGE patterns previously noted amongst SS isolates [12]. Given that phages appear to play such a prominent role in the diversification and evolution of O157 and other *E*. *coli*, it is tempting to speculate that they may play a role in development of the SS phenotype—perhaps through the transfer of a specific adaptive trait or regulatory mechanism that improves adherence or general fitness of the organism to replicate in the bovine intestinal tract. This hypothesis may be testable through additional comparative genomic characterization of phages amongst SS and other O157 isolates.

Finally, the results of our studies reveal several SNPs that differentiate SS strains from each other and from reference O157 isolates. Further, the patterns of distribution of these SNPs provide evidence for positive selection/adaptation of genes/traits involved in the virulence and adaptation of O157 to a life associated with a host—for instance, through shared non-synonymous substitutions in genes relating to adherence, iron acquisition, and motility. Given the distinctive hyper-adherent phenotype for RSE cells that is shared by SS strains, studies are needed to investigate the potential role of key virulence related loci identified to have variation through our current comparative genomics studies and how this may contribute to the SS phenotype and increased fitness of the organism.

In conclusion, our current comparative genomics studies of SS isolates have provided key insights into the overall genomic structure and patterns of evolution of SS strains of O157, as well as lay a strong foundation for future investigations on the microbial mechanisms that may drive super-shedding of this important food-borne pathogen.

## Supporting information

S1 Table PHAST analysis of the phage region in SS and reference strains. (XLSX)

S2 Table Summary of phage region covering areas of 800kb to 1.8Mb region. (XLSX)

S3 Table Phage encoded region 8 in SS17. (XLSX)

S4 Table Phage encoded CDS in SS52. (XLSX)

S5 Table Comparison of phage region 7 in SS17 with phage region 8 in SS52. (XLSX)

S6 Table nsSNP between SS52 and reference strains of O157 in genes related to virulence, adherence, and metabolism. (XLSX)

S1 Fig Comparative analysis of SS strains and reference O157 genomes. (A) Dendrogram representing cluster analysis of SS isolates based on PFGE patterns as previously described [12] highlighting the two representative isolates, SS17 and SS52, that have been completely sequenced; (B) Circular genome representations of SS17 and SS52. Larger circles depict chromosomal DNA with smaller circles representing plasmids. The blue circles represent the ORFs and the outer circle (purple) represents the phages in each genome; (C) Cladogram based on whole genome alignment reveals that SS17 and SS52 clusters closely with lineage I/II “spinach” outbreak isolates (EC4115 and TW14359) as compared with lineage I outbreak isolates (Sakai and EDL933) or the bovine lineage II isolates; (D) Whole genome alignments of SS strains with reference O157 strains using progressiveMauve depicting 8 homology blocks of similarity and patterns of divergence among the strains.
(TIF)

S2 Fig Adherence patterns of eight additional SS strains of O157 (SS7, SS12, SS27, SS42, SS52, SS67, SS77, SS131) on RSE cells. The strong aggregative adherence is common to all tested supershedder strains. All slides are at 40x magnification, bacteria (green), with RSE cytokeratins (red), and nuclei of cells (blue).
(DOCX)
