# Supplementary figures and images for: Comparative genomics of two super-shedder isolates of Escherichia coli O157:H7
Source: PLoS One. 2017 Aug 10;12(8):e0182940. doi: 10.1371/journal.pone.0182940 (PMC5552260; doi:10.1371/journal.pone.0182940)

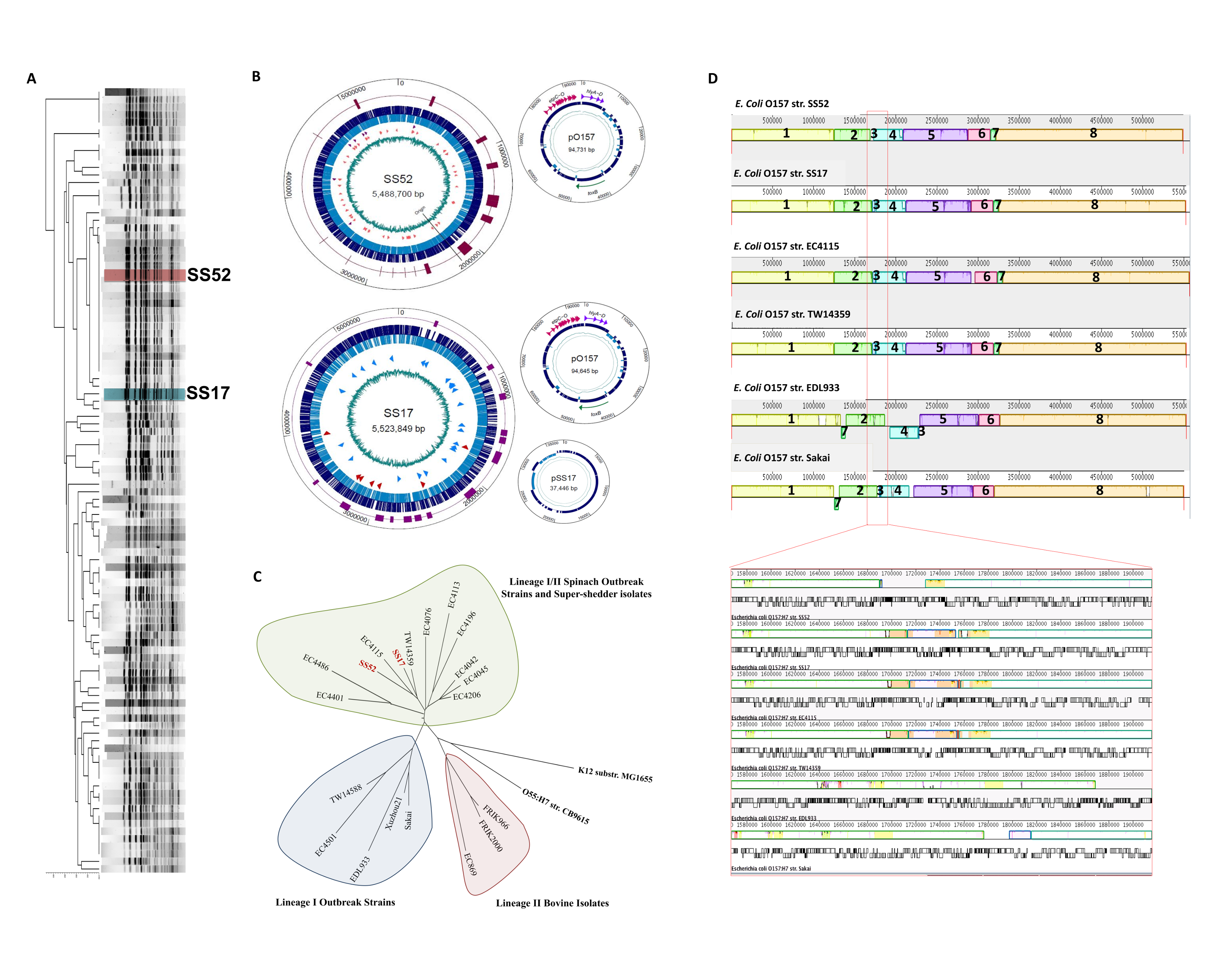

Supplement: S1 Fig — (A) Dendrogram representing cluster analysis of SS isolates based on PFGE patterns as previously described [12] highlighting the two representative isolates, SS17 and SS52, that have been completely sequenced; (B) Circular genome representations of SS17 and SS52. Larger circles depict chromosomal DNA with smaller circles representing plasmids. The blue circles represent the ORFs and the outer circle (purple) represents the phages in each genome; (C) Cladogram based on whole genome alignment reveals that SS17 and SS52 clusters closely with lineage I/II “spinach” outbreak isolates (EC4115 and TW14359) as compared with lineage I outbreak isolates (Sakai and EDL933) or the bovine lineage II isolates; (D) Whole genome alignments of SS strains with reference O157 strains using progressiveMauve depicting 8 homology blocks of similarity and patterns of divergence among the strains. (TIF) [file pone.0182940.s007.tif]

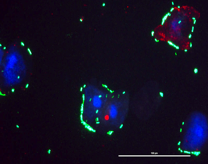


SS42


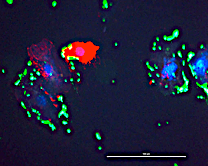


SS27


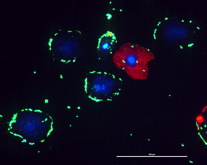


SS12


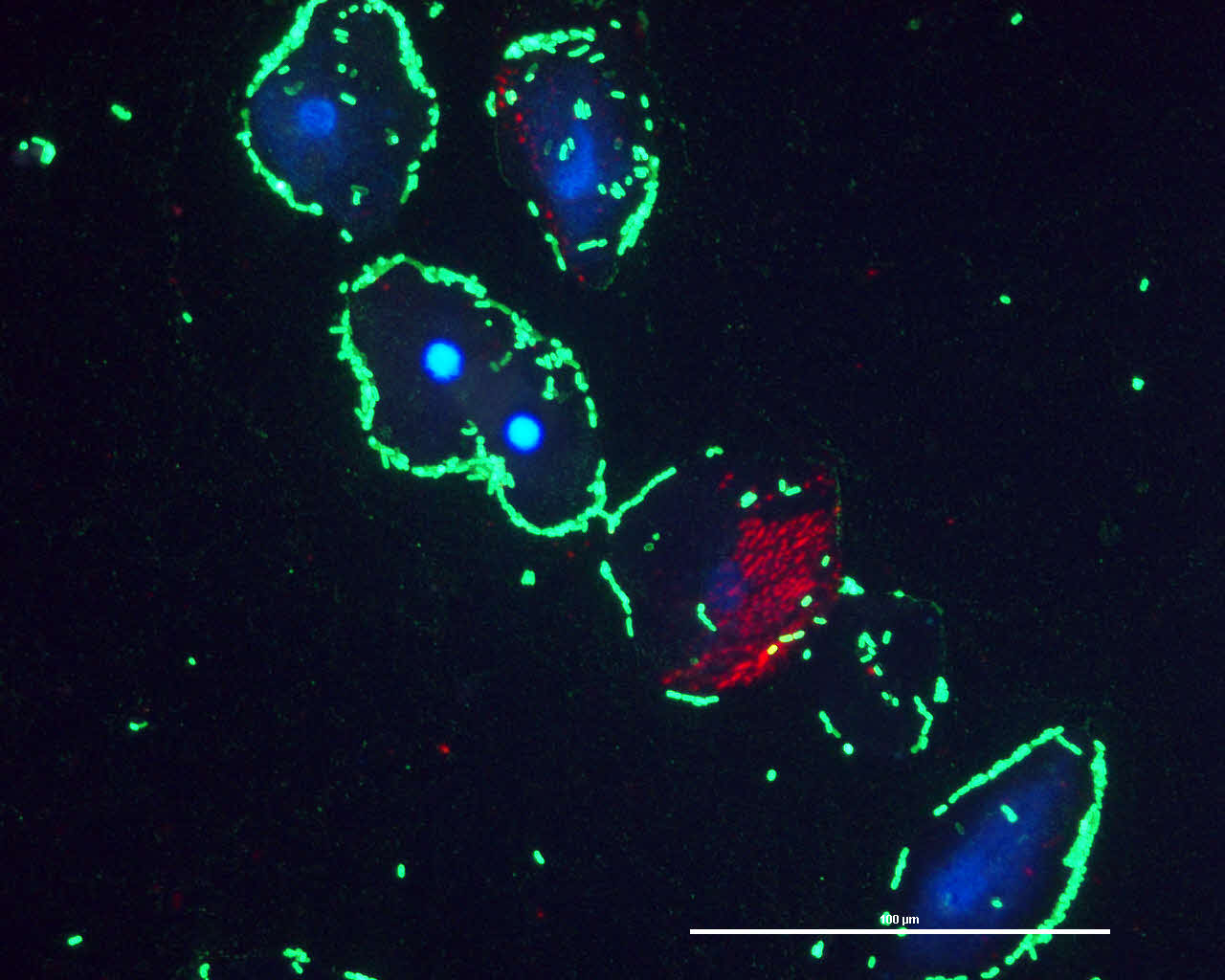


SS7


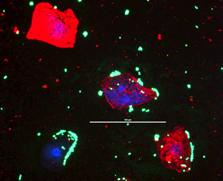


SS52


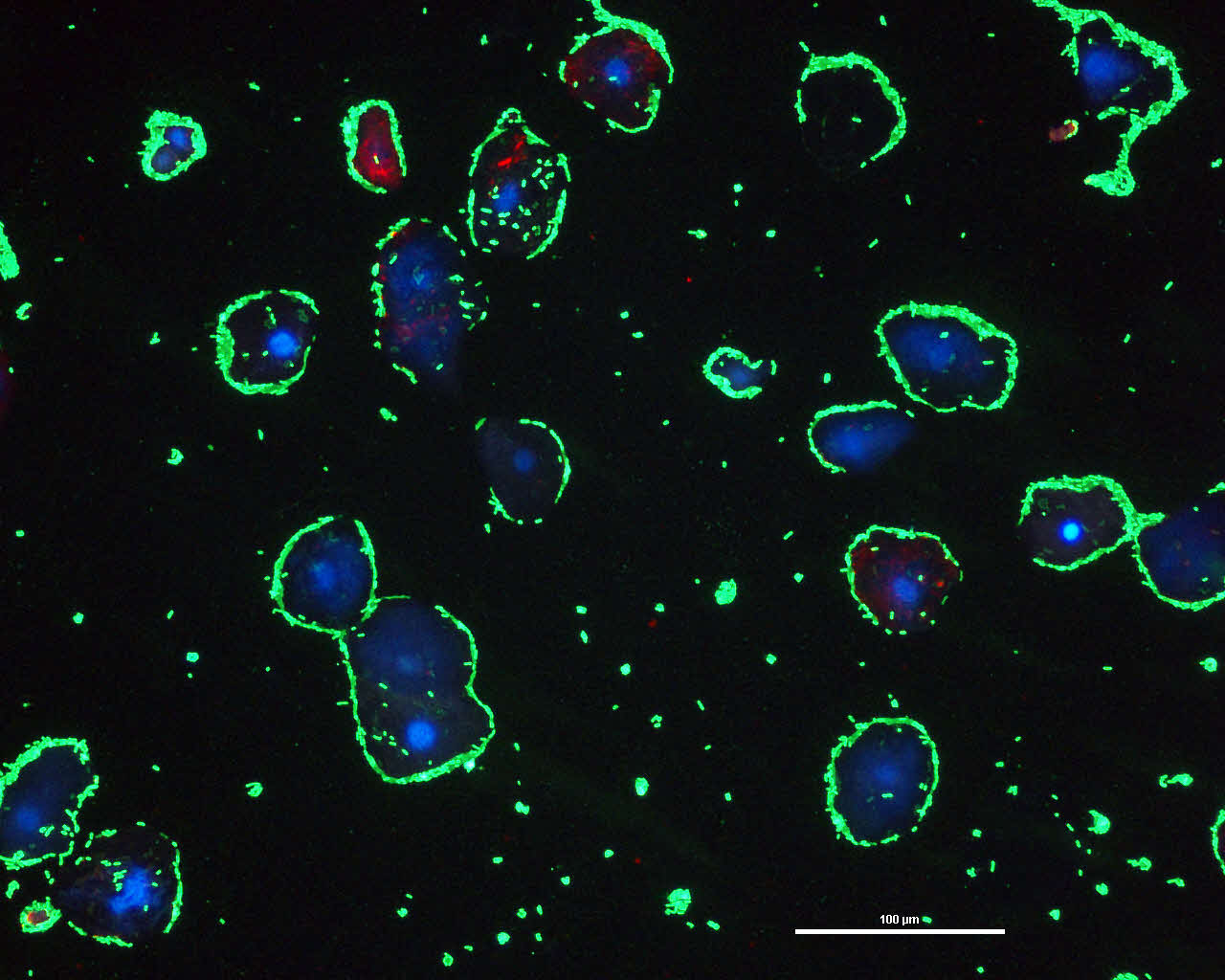


SS131


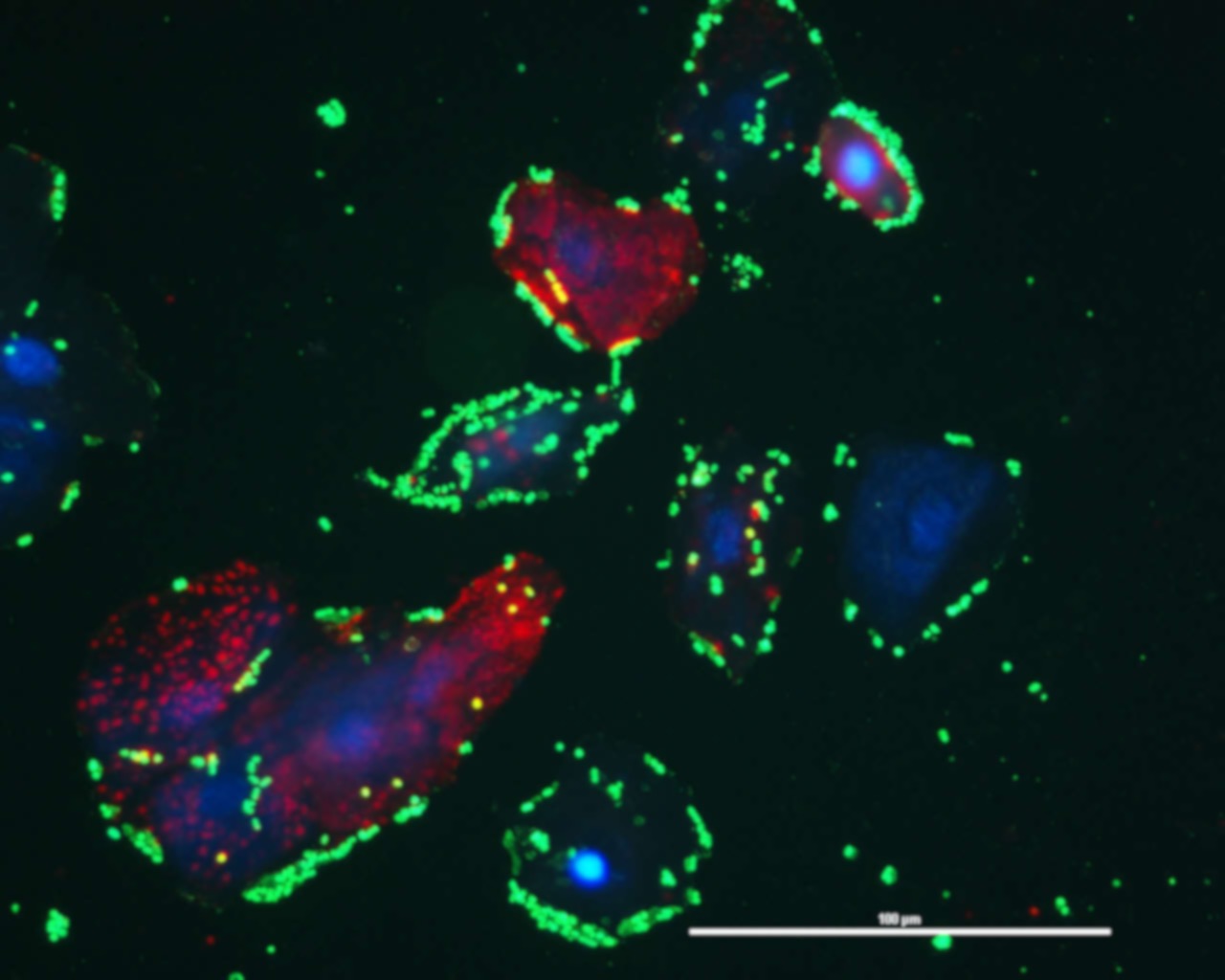


SS77


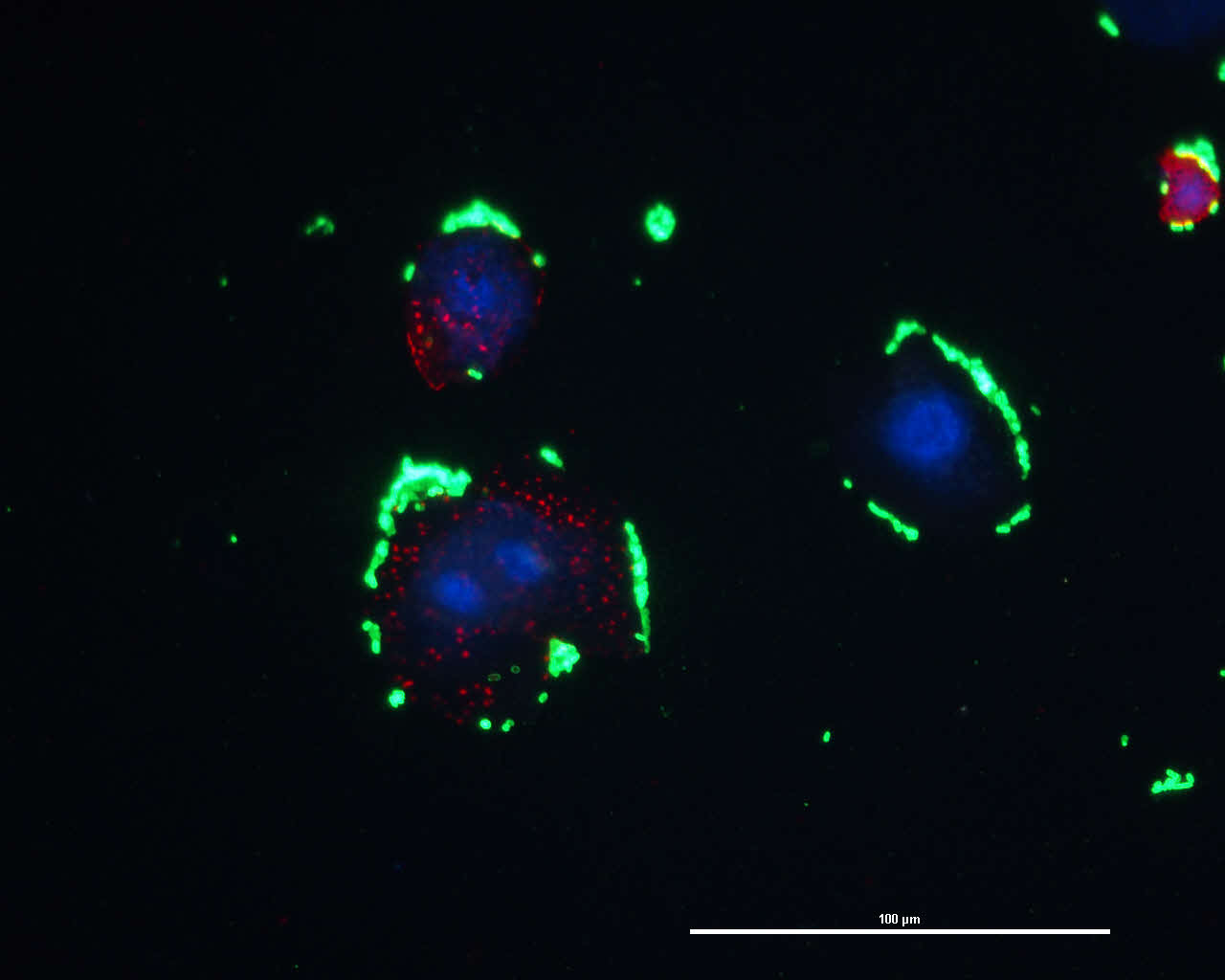


SS67

Supplement: S2 Fig — The strong aggregative adherence is common to all tested supershedder strains. All slides are at 40x magnification, bacteria (green), with RSE cytokeratins (red), and nuclei of cells (blue). (DOCX) [file pone.0182940.s008.docx]
